# Barcoding heat shock proteins to human diseases: looking beyond the heat shock response

**DOI:** 10.1242/dmm.014563

**Published:** 2014-04

**Authors:** Vaishali Kakkar, Melanie Meister-Broekema, Melania Minoia, Serena Carra, Harm H. Kampinga

**Affiliations:** 1 University Medical Center Groningen, University of Groningen, Department of Cell Biology, A. Deusinglaan 1, 9713 AV, Groningen, The Netherlands; 2 Università degli Studi di Modena e Reggio Emilia, Dipartimento di Scienze Biomediche, Metaboliche e Neuroscienze, via G. Campi 287, 41125 Modena, Italy.

**Keywords:** Chaperonopathies, Heat shock protein, Protein-aggregation diseases

## Abstract

There are numerous human diseases that are associated with protein misfolding and the formation of toxic protein aggregates. Activating the heat shock response (HSR) – and thus generally restoring the disturbed protein homeostasis associated with such diseases – has often been suggested as a therapeutic strategy. However, most data on activating the HSR or its downstream targets in mouse models of diseases associated with aggregate formation have been rather disappointing. The human chaperonome consists of many more heat shock proteins (HSPs) that are not regulated by the HSR, however, and researchers are now focusing on these as potential therapeutic targets. In this Review, we summarize the existing literature on a set of aggregation diseases and propose that each of them can be characterized or ‘barcoded’ by a different set of HSPs that can rescue specific types of aggregation. Some of these ‘non-canonical’ HSPs have demonstrated effectiveness *in vivo*, in mouse models of protein-aggregation disease. Interestingly, several of these HSPs also cause diseases when mutated – so-called chaperonopathies – which are also discussed in this Review.

## Introduction

Many heat shock protein (HSP) family members are known to function as molecular chaperones, meaning that they stabilize and assist in the correct folding of nascent polypeptides (Ellis and Hartl, 1999). In addition to their role in *de novo* protein folding, HSPs are involved in various aspects of proteome maintenance, including macromolecular-complex assembly, protein transport and degradation, as well as aggregate dissociation and refolding of stress-denatured proteins. Under normal cellular conditions, HSP levels match the overall level of protein synthesis. Under conditions of stress, mature proteins unfold and exceed the capacity of chaperone systems to prevent aggregation. Such acute proteotoxic stress induces a regulated response resulting in increased expression of some HSPs, which helps to rebalance protein homeostasis.

The human genome encodes more than 100 different HSPs, which are grouped into seven different families: HSPH (Hsp110), HSPC (Hsp90), HSPA (Hsp70), DNAJ (Hsp40), HSPB [small Hsp (sHsp)], the human chaperonins HSPD/E (HSP60/HSP10) and CCT (TRiC), plus several regulatory co-factors (Kampinga et al., 2009). In terms of their regulation, the HSP family members can also be categorized into three groups: (1) constitutively expressed, but not induced by stress; (2) constitutively expressed and induced upon stress; and (3) induced only upon stress (Morimoto, 2008). In addition to their differential regulation, the various HSPs also show a large degree of functional diversity with respect to client specificity and client processing (Kampinga and Craig, 2010). These functional differences could be very important when investigating their potential relevance for diseases in which cells are chronically exposed to proteins that are prone to form toxic protein aggregates. Examples of such diseases are polyglutamine (polyQ) diseases, Parkinson’s disease (PD), amyotrophic lateral sclerosis (ALS) and Alzheimer’s disease (AD). This Review discusses how these diseases can be labeled or ‘barcoded’ by specific sets of HSPs that can rescue their disease-specific aggregations.

## The cellular functions of HSPs

### HSPs and *de novo* protein folding

**Fig. 1.**
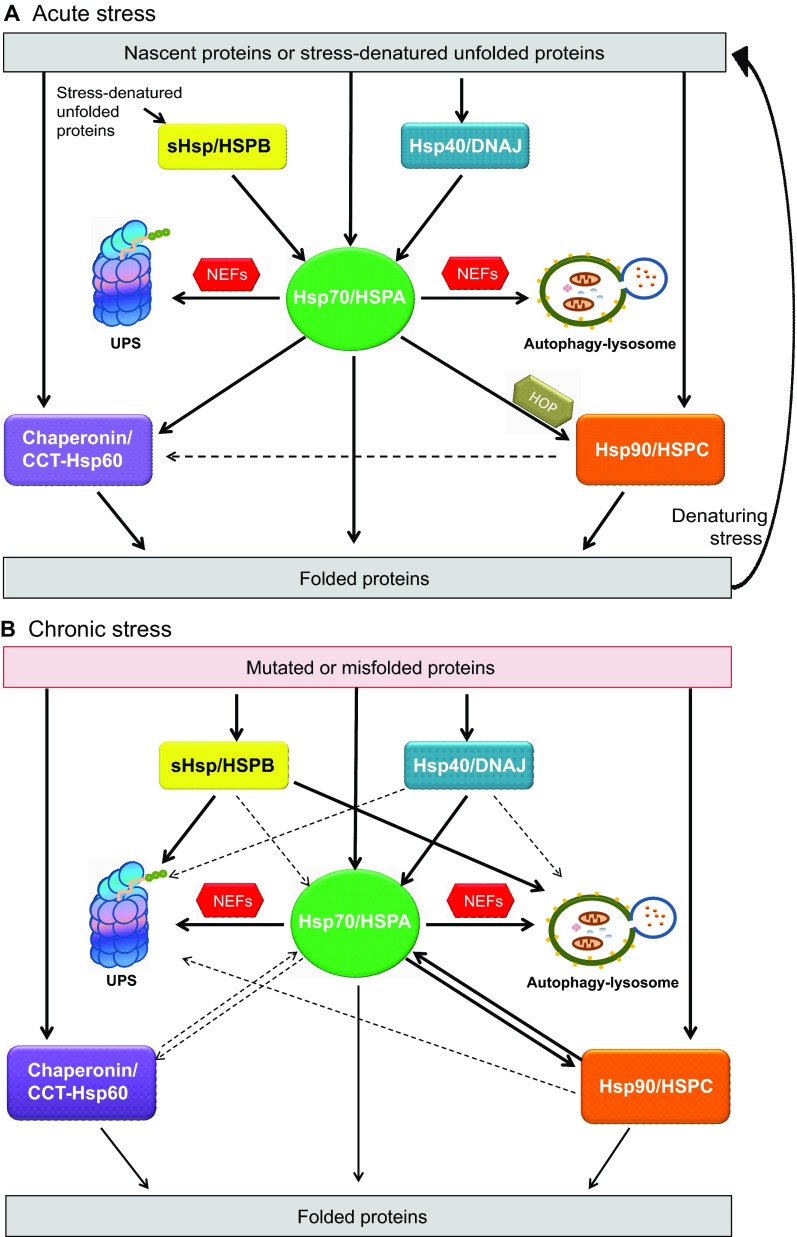
**Model of actions and interactions of the HSP network required for normal protein folding and refolding upon acute stress or during chronic stress.** HSP families constitute a large group of chaperones that interact with non-native proteins, assisting their correct protein folding. HSPs are constitutively expressed, but their expression levels can increase under conditions of stress. They are mainly divided into groups: sHsp/HSPBs, Hsp70/HSPAs, Hsp90/HSPCs and members of the chaperonin (CCT-Hsp60) family (see main text for details). (A) During *de novo* protein folding and for the refolding of acute-stress-denatured unfolded proteins, the functional cooperation of different HSPs is primarily aimed at the structural stabilization of native proteins for (re)folding. However, in case of failure of protein folding, HSPs can also assist client degradation through the ubiquitin-proteasome system (UPS) or the autophagy-lysosome pathway. The central component of the chaperone network and folding catalysts is the Hsp70/HSPA family. Hsp40/DNAJs hydrolyze ATP (bound to Hsp70/HSPA) to ADP, increasing the affinity of its substrate-binding domain for unfolded proteins. Nucleotide-exchange factor (NEF) proteins remove ADP and substitute ATP, reducing Hsp70/HSPA’s substrate-binding affinity, allowing release of the folded protein. Proteins that are unable to utilize Hsp70/HSPAs for complete folding are transferred to the chaperonin or the Hsp90/HSPC system. For transfer of substrates from Hsp70/HSPA to Hsp90/HSPC, HOP is required as a co-chaperone. Under acute stress conditions, HSPB oligomers dissociate into dimers to bind unfolded substrates, thereby avoiding irreversible aggregation of client proteins. This process allows ATP-dependent chaperones to assist in the substrates refolding when normal physiological conditions are restored. (B) In the presence of chronic stress, which triggers protein misfolding, re-folding attempts might be particularly unsuccessful. Under such conditions, the HSP network can assist in protein unfolding and disaggregation, and specific targeting of the misfolded or even aggregated proteins for degradation is usually required. Members of each HSP family are shown to interact with misfolded proteins and to reverse the formation of aggregates. However, whether different HSPs functionally cooperate with each other in order to modulate mutated protein toxicity is not yet clear. Solid lines indicate confirmed actions and interactions; hashed lines refer to those that are suggested but not fully proven.

The general organization of co-translational folding is highly conserved throughout evolution. Ribosome-binding chaperones (e.g. specialized Hsp70/HSPAs) first interact with the nascent polypeptide, followed by a second set of HSPs that do not have a direct affinity for the ribosome (the classical Hsp70/HSPA system). The Hsp70/HSPA family is the central component of the cellular network of molecular chaperones and folding catalysts (Fig. 1A). Hsp70/HSPA proteins are involved in a wide range of protein quality control (PQC) functions, including *de novo* protein folding, refolding of stress-denatured proteins, protein transport, membrane translocation and protein degradation. Hsp70/HSPAs never function alone; they require Hsp40/DNAJ proteins and nucleotide-exchange factors (NEFs) as partners. DNAJ proteins bind and deliver client proteins to the Hsp70/HSPA system, upon which the client protein and DNAJ function together to stimulate HSPA to hydrolyze ATP, leading to high substrate affinity of HSPA. Following ATP hydrolysis, NEFs such as BAG-1, HSPBP1 and HSPH bind HSPA and induce ADP-ATP exchange, leading to substrate release. DNAJs thus mainly confer client specificity to the Hsp70/HSPA machine, but can also affect the fate of HSPA clients, whereas NEFs seem to be mainly involved in client fate (Bukau et al., 2000; Kampinga and Craig, 2010; Young, 2014) (Fig. 1A). The DNAJ/HSPA system might also receive clients from small Hsp/HSPB proteins. HSPB chaperone activity does not need ATP. However, direct interaction with ATP-dependent chaperones such as HSPA promotes the release of the bound substrate and subsequent refolding (Boncoraglio et al., 2012; Garrido et al., 2012).

Proteins that cannot be completely folded by Hsp70/HSPA machines are transferred to, or handled independently by, the chaperonins or the Hsp90/HSPC system (Buchner, 1999; Yam et al., 2008) (Fig. 1A). Substrate transfer to Hsp90/HSPC protein is mediated by the HSP-organizing protein (HOP), which uses multiple tetratricopeptide-repeat domains to form a bridge between HSPA and HSPC (Buchner, 1999; Young et al., 2001). The mechanism of handover from Hsp70 to chaperonins remains unclear in mammals; however, work in the prokaryotic system has begun to reveal some interesting possibilities. For example, it has recently been shown that the Hsp70 homolog DnaK binds the M domain of ClpB to recruit DnaK-bound substrates to the chaperonin (Seyffer et al., 2012).

### HSPs and acute proteotoxic-stress conditions

Cells are constantly challenged by changes in their environment. Acute stress conditions such as heat shock cause many proteins to become unfolded. The accumulation of stress-denatured proteins increases the risk of aggregate formation. In addition to their role in co-translational folding, the constitutively expressed HSP members might also assist in aggregate protection and refolding of stress-unfolded proteins (Fig. 1A). However, it has been shown in yeast that the stress-inducible cytosolic members of the families, which are strongly upregulated by the transcription factor heat shock factor-1 (HSF-1), become more important under such conditions (Albanèse et al., 2006). Next to this transcriptional response, HSPB proteins represent an even more rapid response to environmental stresses (Fig. 1A). Several HSPB members are rapidly and transiently phosphorylated, whereby their oligomeric state is dynamically altered and their protective activities are activated. These protective activities include prevention of cytoskeletal collapse and chaperoning of soluble proteins, which can enhance protein refolding or support client degradation (Garrido et al., 2012).

In parallel to the HSF-1-regulated heat shock response (HSR) in the cytosol, interconnected pathways in different cellular compartments also respond to acute cellular stress, including the unfolded protein response (UPR) in the endoplasmic reticulum and the mitochondria (Haynes and Ron, 2010; Morimoto, 2011; Walter and Ron, 2011). Each pathway not only induces the transcriptional upregulation of genes that enhance refolding capacity, but also the expression of HSP members that assist in degradation of unfolded proteins through the proteasome- and lysosome-mediated pathways, together protecting cells from stress (Parsell and Lindquist, 1993; Haynes and Ron, 2010; Morimoto, 2011; Walter and Ron, 2011).

### HSPs and chronic stress conditions

Protein aggregation hallmarks a high number of chronic diseases (Balch et al., 2008) that can either be loss-of-function or toxic gain-of-function disorders. Loss-of-function diseases, including cystic fibrosis and Gaucher’s disease, are typically caused by recessive mutations that lead to inefficient folding of the mutated proteins and their consequent degradation or dysfunction (Fan et al., 1999) (Fig. 1B). Of note, in recessive diseases, the HSF-1-regulated HSPs can promote some refolding of (metastable) mutant proteins, thereby displaying disease-rescuing potential (Yang et al., 2013). In addition, chaperone inhibition, resulting in less efficient recognition of the mutant peptides and therefore increasing their degradation, has been shown to be protective in such diseases (Chanoux and Rubenstein, 2012). Toxic gain-of-function diseases, on the other hand, usually manifest with the formation of intracellular and/or extracellular deposits of aggregated proteins, as will be further discussed below (Chiti and Dobson, 2006; Balch et al., 2008; Morimoto, 2008). These aggregates are often fundamentally different from those formed during acute stress because they initially are formed without being sensed by the (acute) stress responses in the cells. Moreover, unlike in response to acute stress, in which proteins are unfolded, proteins in chronic stress are intrinsically misfolded and can generally not be refolded; these misfolded proteins must be disposed of (Fig. 1B). This could imply that different HSPs might be crucial – or rate-limiting – to providing protection in chronic protein-aggregation diseases than for acute stress. Below, we will focus on toxic gain-of-function diseases and provide an overview of the literature on HSPs that could prevent aggregation or/and toxicity of the disease-associated proteins. Because we aim to identify HSPs that might be rate-limiting factors for aggregate prevention and thus targets for intervention in these diseases, we will mainly discuss effects of HSP overexpression and not include studies on the downregulation of HSPs. Interestingly, HSP downregulation is often associated with toxicity and lethality and can result in disease itself. Therefore, this Review will also provide an overview of aggregation diseases, known as chaperonopathies, which are caused by mutations in HSPs. In this way, we aim to recapitulate the role of HSPs in chronic aggregation diseases from two angles: the prevention of toxic gain-of-function diseases and their role in causing disease themselves.

## HSPs and proteinopathies

**Fig. 2.**
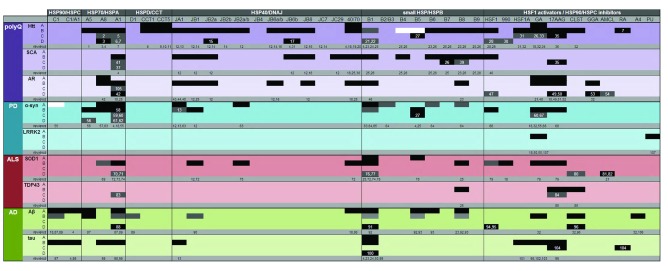
**HSP barcodes associate with diverse proteinopathies.** Summary of literature pertaining to the effects on proteinopathies of activating either the cytosolic heat shock response (HSR/HSF-1), using HSF-1 activators or HSP90 inhibitors, or overexpressing specific HSPs from the different families (HSPC, HSPA, HSPD/CCT, DNAJ or HSPB). For each disease, evidence was categorized into four levels according to the system or organism in which the effect was examined: *in vitro* (A), cell studies (B), non-mammalian model systems (C) and mammals (D). Evidence was further graded according to the specific effects of the HSP(s) on the disease: prevention of aggregate formation (black), buffering of toxic effects caused by diseased protein (gray) and absence of effects (white). See main text for further explanation. Numbers in the table correspond to references in the legend. Solid data was cited from review articles, which are displayed as numbers in the gray column below each disease section. Data from orginial articles (higher model organisms, subheadings C and D) are cited in the corrseponding individual cells. *reviews*, articles with general information used for the figure; polyQ, polyglutamine diseases; Htt, huntingtin; SCA, spinocerebellar ataxia; AR, androgen receptor; PD, Parkinson’s disease; α-syn, α-synuclein; ALS, amyotrophic lateral sclerosis; AD, Alzheimer’s disease; Aβ, amyloid-β; 990, HSP990; AMCL, arimoclomol; GA, geldanamycin; CLST, celastrol; GGA, geranylgeranylacetone; RA, radicicol; A4, drug name (novobiocin analog); PU, PU-H71. References: (1) Jiang et al., 2012; (2) Gunawardena et al., 2003; (3) Bauer et al., 2010; (4) Yousuf et al., 2010; (5) McLear et al., 2003; (6) Hansson et al., 2003; (7) Hay et al., 2004; (8) Tam et al., 2006; (9) Sontag et al., 2013; (10) Tam et al., 2009; (11) Behrends et al., 2006; (12) Kakkar et al., 2013; (13) Abisambra et al., 2012; (14) Hageman et al., 2011; (15) Labbadia et al., 2012; (16) Hageman et al., 2010; (17) our unpublished results; (18) Peterson and Blagg, 2009; (19) Jana et al., 2000; (20) Wacker et al., 2004; (21) Perrin et al., 2007; (22) Zourlidou et al., 2007; (23) Carra and Landry, 2006; (24) Mymrikov et al., 2011; (25) Boncoraglio et al., 2012; (26) Vos et al., 2010; (27) Tue et al., 2012; (28) Fujimoto et al., 2005; (29) Pierce et al., 2010; (30) Labbadia et al., 2011; (31) Neef et al., 2010; (32) Neef et al., 2011; (33) Agrawal et al., 2005; (34) Sittler et al., 2001; (35) Fujikake et al., 2008; (36) Herbst and Wanker, 2007; (37) Cummings et al., 2001; (38) Chai et al., 1999; (39) Carra et al., 2010; (40) Rimoldi et al., 2001; (41) Chan et al., 2000; (42) Adachi et al., 2003; (43) Fliss et al., 1999; (44) Stenoien et al., 1999; (45) Howarth et al., 2007; (46) Stope et al., 2012; (47) Kondo et al., 2013; (48) Thomas et al., 2006; (49) Waza et al., 2005; (50) Waza et al., 2006; (51) Almeida et al., 2011; (52) Rusimini et al., 2011; (53) Katsuno et al., 2005; (54) Malik et al., 2013; (55) Aridon et al., 2011; (56) Gorbatyuk et al., 2012; (57) Redeker et al., 2012; (58) Danzer et al., 2011; (59) Auluck et al., 2002; (60) Auluck et al., 2005; (61) Klucken et al., 2004; (62) Shimshek et al., 2010; (63) Pemberton et al., 2011; (64) Bruinsma et al., 2011; (65) Outeiro et al., 2006; (66) Liangliang et al., 2010; (67) Auluck and Bonini, 2002; (68) Riedel et al., 2010; (69) Song et al., 2013; (70) Gifondorwa et al., 2007; (71) Gifondorwa et al., 2012; (72) Boillée et al., 2006; (73) Koyama et al., 2006; (74) Patel et al., 2006; (75) Blumen et al., 2012; (76) Sharp et al., 2008; (77) Krishnan et al., 2008; (78) Yerbury et al., 2013; (79) Batulan et al., 2006; (80) Kiaei et al., 2005; (81) Kalmar et al., 2008; (82) Kieran et al., 2004; (83) Estes et al., 2011; (84) Gregory et al., 2012; (85) Jinwal et al., 2012; (86) Evans et al., 2006; (87) Tiffany-Castiglioni and Qian, 2012; (88) Hoshino et al., 2011; (89) Veereshwarayya et al., 2006; (90) Carnini et al., 2012; (91) Toth et al., 2013; (92) Wilhelmus et al., 2006; (93) Wilhelmus et al., 2007; (94) Jiang et al., 2013; (95) Pierce et al., 2013; (96) Paris et al., 2010; (97) van der Putten and Lotz, 2013; (98) Dou et al., 2003; (99) Miyata et al., 2011; (100) Abisambra et al., 2010; (101) Opattova et al., 2013; (102) Petrucelli et al., 2004; (103) Dickey et al., 2006; (104) Sinadinos et al., 2013; (105) Chan et al., 2002; (106) Ansar et al., 2007; (107) Wang et al., 2008.

There are numerous human diseases that are associated with the aggregation of a single dominant peptide or protein. Examples of such diseases, known as proteinopathies, include polyQ diseases, PD, ALS and AD. The monogenic forms of neurodegenerative proteinopathies are rare, and are generally histopathologically indistinguishable from their corresponding sporadic forms, making it likely that both forms share a final common pathway. Protein aggregates are either found inside neurons (e.g. tau tangles in AD) or outside neurons, in the extracellular space [e.g. amyloid-β (Aβ) plaques in AD]. Aggregates are generated when proteins become destabilized, either by mutations changing their native state (e.g. SOD1 in ALS) or quantity (e.g. α-synuclein in PD), by the elongation of a certain domain [e.g. huntingtin (Htt) in Huntington’s disease (HD)] or by domain truncations (e.g. TDP-43 in ALS). Aggregates range from extremely dense amyloidogenic aggregates with β-sheet cores (Htt, ataxin-3, Aβ) to more amorphous aggregates (α-synuclein, SOD1, TDP-43). Although it is still debated whether the small oligomers or the large inclusions are more toxic, the overall evidence from model systems strongly suggests that aggregate prevention generally results in disease amelioration. Therefore, this Review focuses on aggregate prevention by HSPs and will reveal that each of these proteinopathies is associated with a different pattern or ‘barcode’ of rescue depending either on the HSR or individual HSPs. The elucidation of these barcodes provides a platform for a rational design of disease-specific therapeutic strategies. For each disease, we have categorized evidence into four levels (Fig. 2): *in vitro* (lowest level), cell studies, non-mammalian model systems and mammals (highest level). Furthermore, evidence in Fig. 2 was graded according to the specific effects of each chaperone: prevention of aggregate formation (black), buffering of toxic effects caused by diseased protein (gray) and absence of protective effects (white).

### Polyglutamine (polyQ) diseases

In polyQ diseases, the polyQ tract is elongated beyond a certain threshold. The transcribed polyQ peptide fragments are thought to be the initiators of amyloid fibrils and have a strong propensity to assemble into highly ordered polymers that are extremely rich in β-sheet structure, thereby creating sodium dodecyl sulfate (SDS)-insoluble aggregates (Wellington et al., 2000; Chiti and Dobson, 2009). PolyQ expansions in Htt, ataxins and the androgen receptor have been associated, respectively, with the dominant late-onset toxic gain-of-function diseases HD, spinocerebellar ataxias (SCA) and spinal bulbar muscular atrophy (SBMA). All these diseases are associated with severe motor problems and/or muscle atrophy (Chiti and Dobson, 2009; Banno et al., 2012; Seidel et al., 2012b). Both age of onset and protein-aggregation propensity are strongly associated with the length of the polyQ expansion, further suggesting that aggregate formation forms the basis of disease (Gusella and MacDonald, 2000; Wellington et al., 2000).

In cells and non-mammalian model organisms, activation of the acute HSR pathways has been shown to reduce the extent of polyQ aggregation. Overexpression of HSF-1 leads to fewer but larger polyQ aggregates in cells (Pierce et al., 2010). In agreement with this finding, chemical upregulation of the HSR in cells and nonmammalian animal organisms reduced a number of dysfunctions caused by polyQ overexpression (see Fig. 2 for associated references). Although overexpression of HSF-1 in muscle tissue of the R6/2 mouse model for HD increased lifespan, there were only small effects on aggregates (Fujimoto et al., 2005), implying that effects were compensatory and did not affect the underlying toxic gain of function. Chemical upregulation of the HSR by the use of Hsp90/HSPC inhibitors in the R6/2 mouse model leads to transient beneficial effects, which disappear during disease progression (Labbadia et al., 2011). Moreover, Hsp90/HSPC inhibition leads to accelerated degradation of soluble polyQ-Htt, which is apparently independent of HSR activation; however, this was most likely due to pleiotropic effects associated with the inhibition of Hsp90/HSPC instead (Baldo et al., 2012; Yam et al., 2008; Buchner, 1999).

Whereas injection of HSF-1 into an SBMA mouse model resulted in only small effects in neurons, Hsp90/HSPC inhibition by 17-AAG, geranylgeranylacetone (GGA) and geldanamycin (GA) not only substantially increased lifespan, but also diminished aggregates (Fig. 2). Hsp90s/HSPCs are required for the degradation, regulation, ligand-binding affinity and stabilization of the androgen receptor, as well as for its trafficking (Peterson and Blagg, 2009). The strong effects of Hsp90/HSPC inhibitors on SBMA – but not HD – suggest that HSF-1 activation and the resulting upregulation of the HSR is insufficient to modulate polyQ diseases in general. Protective effects of Hsp90/HSPC inhibitors, if found, are therefore most likely due to HSF-1-unrelated effects (Baldo et al., 2012).

Upregulation of individual members from HSF-1-regulated HSP families (e.g. HSPA1A, DNAJB1, HSPB1) was effective in preventing polyQ aggregation or the associated toxicity *in vitro* and in cellular models (Fig. 2). However, in comparative screens involving larger polyQ expansions, the HSR-regulated HSPs were usually rather ineffective compared with non-canonical HSPs (Vos et al., 2010; Hageman et al., 2011; Hageman et al., 2012). Some effects of Hsp70/HSPA overexpression on polyQ toxicity were reported in *Drosophila melanogaster* (Fig. 2). However, these effects were not associated with aggregate reduction, suggesting that the observed protection was due to compensatory effects downstream of aggregate formation; for instance, the loss of normal PQC functions owing to entrapment of key chaperones, such as DNAJB1 (Park et al., 2013). Yet, this loss of PQC is apparently not at the heart of disease in mammals, because restoration of PQC by HSP70 overexpression did not delay disease progression in the R6/2 HD mouse model (Hansson et al., 2003; Hay et al., 2004). The same is true for the canonical small HSP HSPB1. Although an earlier report suggested that HSPB1 overexpression led to a small delay of Htt toxicity in rats (Perrin et al., 2007), studies in a mouse model for HD (Zourlidou et al., 2007) as well as several studies in cells (Fig. 2) showed that HSPB1 is rather inefficient in delaying polyQ aggregation. Combining these results suggests that HSPB1 might have some compensatory effects that might initially slightly delay disease but, because HSPB1 seems not to affect aggregates directly, this is insufficient to substantially rescue the disease in mammals (Fig. 2).

In dedicated screens for members of the HSP families that might be better suppressors of polyQ aggregation, a number of very effective HSPs were identified, including DNAJB2, DNAJB6, DNAJB8, HSPB6, HSPB7, HSPB8 and HSPB9. Interestingly, most of these were not, or were only marginally, regulated by HSF-1 and were not effective in stimulating substrate refolding after acute stress (Vos et al., 2010; Hageman et al., 2011; Hageman et al., 2012; Kakkar et al., 2013). Instead, these HSPs were associated with degradation of clients through the proteasomal and autophagic degradation routes. Moreover, whereas DNAJB6, HSPB7 and HSPB8 delayed aggregation in *Drosophila*, DNAJB2 was the first HSP that demonstrated a protective effect on aggregate formation, functional end points and survival in mice (Labbadia et al., 2012). Interestingly, our preliminary data regarding transgenic overexpression of DNAJB6 indicate even larger protective effects in the R6/2 mice (our unpublished results). The effectiveness of these non-canonical HSPs in cells, non-mammalian model organisms and mice might be related to their ability to prevent initiation of aggregate formation or to assist aggregate clearance through autophagy, a finding that would be consistent with the important role that autophagy plays in proteinopathies (Vos et al., 2010; Boncoraglio et al., 2012; Rubinsztein et al., 2012; Gillis et al., 2013; Mansson et al., 2013).

In a nutshell, the HSR and individual HSF-1-regulated HSP members have marginal and mainly compensatory effects in polyQ diseases. In contrast, other members of the HSP families that can prevent aggregate initiation or dispose of aggregates might have potential as targets for therapy in polyQ diseases.

### Parkinson’s disease (PD)

About 5–10% of PD cases are monogenic and are caused by either loss-of-function or toxic gain-of-function mutations. The most commonly occurring PD-causing mutations are in the mitochondria-associated genes encoding Parkin (PARK2), PINK1 (PARK6) and DJ-1 (PARK7) (Lesage and Brice, 2009; Schapira and Tolosa, 2010; Martin et al., 2011; Klein and Westenberger, 2012). Mutations in these genes are recessively inherited and usually result in a loss-of-function effect, mainly impeding mitochondrial function and turnover. By contrast, a toxic gain-of-function phenotype resulting in PD is caused by rare dominantly inherited mutations and multiplications in the genes *SNCA* (*PARK1*, *PARK4*) and *LRRK2* (*PARK8*) (Lesage and Brice, 2009; Schapira and Tolosa, 2010; Klein and Westenberger, 2012). This Review will focus on these rare toxic gain-of-function mutations.

Mutations in or multiplications of *SNCA* lead to increased oligomerization of the gene product α-synuclein, which is an intrinsically disordered protein. This enhanced oligomerization increases the tendency of α-synuclein to form β-sheet structures and eventually fibrous amyloidogenic inclusions, called Lewy bodies and Lewy neurites (Lesage and Brice, 2009; Martin et al., 2011; Roostaee et al., 2013). LRRK2 is a kinase that is involved in the phosphorylation of α-synuclein. Mutations in LRRK2 are thought to promote α-synuclein expression, aggregation and toxicity, thereby increasing the propensity of α-synuclein to self-aggregate (Schapira and Tolosa, 2010; Martin et al., 2011).

As in polyQ diseases, genetic or chemical activation of HSF-1 can temporarily compensate for LRRK2 and α-synuclein toxicity in cells and *Drosophila* (Fig. 2).

Although individual HSPs such as DNAJA1, DNAJB2, HSPB2/HSPB3, HSPB6 and HSPB8 inhibited α-synuclein aggregation *in vitro*, none of them have proven to be effective in this action in cells thus far (Fig. 2). HSPB1 and HSPB5 were found to be effective in preventing α-synuclein aggregation *in vitro*, in cells and in *Drosophila*; however, there is currently no evidence of success in mouse models. Overexpression of HSPA1 was also shown to be able to inhibit α-synuclein aggregation *in vitro*, and decrease α-synuclein toxicity in cells and in *Drosophila*. Moreover, HSPA1 overexpression in mice did show some protective effects, although the data are still disputed (Klucken et al., 2004; Shimshek et al., 2010) (Fig. 2). As is the case for polyQ diseases, neither Hsp70/HSPA1 nor any other canonical HSP could prevent aggregate formation or reduce aggregate size and quantity.

These data taken together would suggest that compensation for loss of normal PQC by sequestration of HSPs into aggregates plays a more important role in PD than it does in polyQ diseases. In line with this notion, a study of α-synuclein in mice showed that transgenic overexpression of HSPA5 delayed disease onset without affecting cytosolic protein aggregation. Because HSPA5 is an ER-resident Hsp70/HSPA and is not expressed in the cytoplasm of the cell, its mode of action must be indirect. Instead of directly affecting aggregate formation, HSPA5 most likely compensates for downstream consequences of aggregation and thereby delays disease onset (Gorbatyuk et al., 2012).

To conclude, the biophysical nature and intracellular localization of α-synuclein aggregates are clearly different from aggregates in polyQ diseases (Ciechanover and Brundin, 2003). Expression of (mutant) α-synuclein rapidly activates HSF-1, whereas polyQ expression either does not activate HSF-1 at all, or only transiently and very late in disease (Ciechanover and Brundin, 2003; Seidel et al., 2012a). The potential HSP suppressors of PD thus seem to differ from that of polyQ diseases, thereby resulting in a different HSP barcode of potential treatment targets (Fig. 2).

### Amyotrophic lateral sclerosis (ALS)

About 5% of ALS cases are currently categorized as dominant monogenic ALS, the most commonly occurring mutations being in SOD1, TDP-43 and FUS (Andersen and Al-Chalabi, 2011; Al-Chalabi et al., 2012). Clinically, sporadic and monogenic ALS are virtually indistinguishable because SOD1- and TDP-43-positive inclusions are present in both forms of the disease, thereby implying a final common pathway (Turnder et al., 2013). About 166 mutations in *SOD1* have been associated with monogenic ALS. Although SOD1 mutations were initially thought to cause disease via a loss of wild-type SOD1 function, SOD1-knockout mice displayed no phenotype (Saccon et al., 2013). Instead, the overexpression of mutant SOD1 leads to disease, implying that the mutant gained a toxic function (Siddique and Deng, 1996; Andersen and Al-Chalabi, 2011). Mutations in SOD1 indeed structurally destabilize the protein, thereby increasing its aggregation propensity, which eventually results in amyloid-fibril formation (Luheshi and Dobson, 2009). Mutations in TDP-43, an RNA-processing protein that usually shuttles between the nucleus and cytoplasm of the cell, render the protein aggregation-prone, which leads to the formation of dense round or filamentous aggregates in the cytoplasm alongside stress granules (Luheshi and Dobson, 2009; Andersen and Al-Chalabi, 2011; Al-Chalabi et al., 2012). Mutations in FUS, another protein involved in RNA metabolism, also result in large globular and elongated cytoplasmic inclusions (Andersen and Al-Chalabi, 2011; Al-Chalabi et al., 2012). Nevertheless, FUS-related ALS is defined as an atypical form because TDP-43-positive aggregates are not part of the pathology; therefore, this Review will not discuss FUS-related ALS.

Treatment of *Drosophila* with the Hsp90/HSPC inhibitor 17-AAG reduced the characteristic ALS eye-degeneration phenotype in a TDP-43 model (Gregory et al., 2012). In addition, treatment of the SOD1-G93A mouse model with 17-AAG not only delayed age of symptom onset, but also increased lifespan (Kieran et al., 2004; Kiaei et al., 2005; Kalmar et al., 2008). However, these protective effects were not reproducible in the SOD1-G37R or the SOD1-G85R mouse model (Chiti and Dobson, 2009; Gifondorwa et al., 2012). These contradictory results indicate that protein aggregation and toxicity mechanisms might depend on the exact kind of mutation, and therefore result in a different barcode of HSPs for each SOD1 mutation.

Regarding the effects of individual HSPs on SOD1 aggregation and toxicity, data in cell lines expressing mutant SOD1 suggest protective effects of HSPA1, DNAJB1, DNAJB2, HSPB1 and HSPB8 (Fig. 2). Furthermore, HSPB8 alleviated TDP-43 aggregation and toxicity in cells and HSPA1A reduced TDP43-associated eye degeneration in *Drosophila* (Estes et al., 2011). The intracranial injection of SOD1-G93A mice with HSPA1 was also protective, whereas long-term effects of HSPB1 overexpression in mice were absent, although this awaits further investigation (Gifondorwa et al., 2007; Krishnan et al., 2008; Sharp et al., 2008; Gifondorwa et al., 2012).

To conclude, except for the aforementioned Hsp90/HSPC inhibitors, none of the discussed HSPs resulted in long-term rescue or had direct effects on SOD1 aggregates (Fig. 2). Moreover, it is not clear whether the effects of the Hsp90/HSPC inhibitors are due to the elevation of HSF-1-regulated HSPs, or whether they are due to the broad effects that these inhibitors exert on cell homeostasis. In summary, the barcode of HSPs that protect against ALS is still very limited.

### Alzheimer’s disease (AD)

The most commonly occurring form of AD is sporadic late-onset AD. In contrast, only about 1–2% of AD cases occur with early onset, and these are due to autosomal-dominant mutations in amyloid precursor protein (*APP*), presenilin 1 (*PSEN1*) or presenilin 2 (*PSEN2*) (Guerreiro et al., 2012). Although intracellular tangles, consisting of hyperphosphorylated tau and extracellular Aβ plaques, are present in both sporadic and monogenic AD, it is unclear how toxicity in AD proceeds. It is disputed as to whether Aβ aggregation leads to cellular stress and results in tau hyperphosphorylation and aggregation (described as the amyloid cascade hypothesis), or whether tau hyperphosphorylation and aggregation precede Aβ accumulation (described as the tau axis hypothesis) (Götz et al., 2011). Here, we will provide an unbiased summary of the effects of HSPs on both Aβ- and tau-related aggregation.

Aβ peptides are the result of APP cleavage via one of two pathways: a non-amyloidogenic pathway that leads to the generation of the most common isoform, Aβ_40_, or an amyloidogenic pathway that results in the generation of Aβ_42_ (Guerreiro et al., 2012). AD-related mutations in APP usually affect the ratio or properties of these different Aβ species (Guerreiro et al., 2012). Similarly, mutations in PSEN1 and PSEN2, which are rate-limiting components of the γ-secretase complex in the amyloidogenic pathway, result in increased generation of the more fibrillogenic Aβ_42_ (Götz et al., 2011; Guerreiro et al., 2012).

HSF-1 injection into an APP rat model increased neuronal health and reduced Aβ-plaque load (Jiang et al., 2013). Similarly, in another study, genetic overexpression of HSF-1 in APP mice diminished soluble Aβ levels (Pierce et al., 2013). In line with these findings, treatment of APP mice with the HSF-1 activator celastrol slightly decreased Aβ-plaque load (Paris et al., 2010).

*In vitro*, it was shown that HSPA1, HSPA5, HSPC1 and HSPA1/DNAJB1, as well as HSPB1, HSPB5, HSPB6 and HSPB8, slow down Aβ aggregation when upregulated individually (Fig. 2). Furthermore, when cells exposed to purified, extracellularly added Aβ were co-incubated with purified DNAJB1, HSPB1, HSPB5 or HSPB8, they were protected against Aβ toxicity (Wilhelmus et al., 2006; Carnini et al., 2012). However, considering that HSPs are intracellular proteins, whereas Aβ plaques are generally considered to be extracellular, the relevance of such findings could be debated. Interestingly, evidence stating that intracellular Aβ aggregation might precede extracellular plaque formation is accumulating, which increases the relevance of findings indicating that HSPs are able to prevent the initiation of aggregation in cells (Hu et al., 2009; Sakono and Zako, 2010). Although there is no cellular data available at present, these findings might explain why transgenic overexpression of HSPA1 and HSPB1 had protective effects in mouse models for Aβ (Hoshino et al., 2011; Tóth et al., 2013). However, it is questionable whether Aβ aggregation was directly affected by HSPA1 overexpression in transgenic mice, or whether the observed protective effects were due to more general compensatory effects of HSPA1 (Hoshino et al., 2011).

Another protein that has been associated with neuronal death in AD is tau. Tau is an unstructured and dynamic protein that is normally involved in stabilization of microtubules, but becomes hyperphosphorylated and detaches from microtubules under conditions of stress. This detachment results in microtubular collapse and in the aggregation of tau into well-ordered and periodic protein deposits (Götz et al., 2011; Mandelkow and Mandelkow, 2012).

In cells, the HSF-1 activator HSF1A reduced tau aggregation by increasing proteasomal degradation of tau (Opattova et al., 2013). Likewise, Hsp90/HSPC-directed drugs, such as geldanamycin, enhanced clearance of tau from cells, thereby reducing its toxicity (Fig. 2). Moreover, the HSF-1 activator radicicol and the GA derivative 17-AAG alleviated tau toxicity in *Drosophila* larvae (Fig. 2).

Multiple HSPs alleviated tau toxicity in cells, including HSPA8, HSPA1, Hsp90s/HSPC, DNAJA1 and HSPB1 (Fig. 2). In addition, HSPB1 rescued behavioral defects in a mouse model for tauopathy (Abisambra et al., 2010). However, there have been no studies to investigate the role of the other HSPs in mammals to date.

To conclude, interpreting the data about the effects of HSPs on AD needs to be done with great caution for two main reasons. Firstly, it is not yet known whether AD is initiated by intracellular (tau or Aβ) or extracellular (Aβ) aggregates. Secondly, although extracellularly added or leaked HSPs might affect toxicity of Aβ plaques, it is still unclear how intracellular overexpression of HSPs can have direct effects on toxicity in AD. These factors currently limit elucidation of the barcode of HSPs for AD.

### Different aggregation diseases have a different HSP barcode

Although all diseases discussed here are toxic gain-of-function aggregation diseases, the barcode of HSPs with protective potential clearly differs depending on the disease (Fig. 2). This variability strongly suggests that the neurodegenerative proteinopathies discussed in this Review are biochemically and biologically distinct. Because all proteinopathies presumably impede on overall protein homeostasis, simply rescuing the overall folding capacity by activation of the complete (acute) HSR (Fig. 1A), or expression of individual components thereof, might lead to some protective effects. However, these effects are generally small and transient, and do not actually affect aggregate formation of the specific diseased proteins themselves. Instead, the effects of the HSR might compensate for entrapment of chaperones and/or other components into aggregates (e.g. certain crucial transcription factors). The underlying toxicity of the aggregates themselves is likely to go beyond these effects on protein homeostasis and will furthermore directly impair other functions, such as axonal transport, organelle dynamics (physical obstruction or cytoskeletal collapse) and membrane integrity. Assuming that aggregation indeed is the reason for toxicity in all these diseases, each proteinopathy requires specific HSPs that either directly prevent aggregation or that recognize early aggregate intermediates and target these for degradation. These HSPs are likely to be found among the ‘non-canonical HSPs’, many of which have not yet been fully explored for each of these diseases.

## Chaperonopathy: the case of ‘sick’ HSPs

So far, we have highlighted how HSPs might act as a first line of defense in preventing proteinopathies. Sometimes, however, HSPs themselves are mutated, leading to pathological conditions termed chaperonopathies (Macario and Conway de Macario, 2002; Macario et al., 2005; Macario and Conway de Macario, 2007). Although the term ‘chaperonopathy’ was initially used to include any condition associated with putative alteration in the expression, post-translational modification or localization of chaperones, this Review will only discuss those diseases in which genetically inherited mutations in the HSPs are the direct causative factor (Table 1).

**Table 1.**
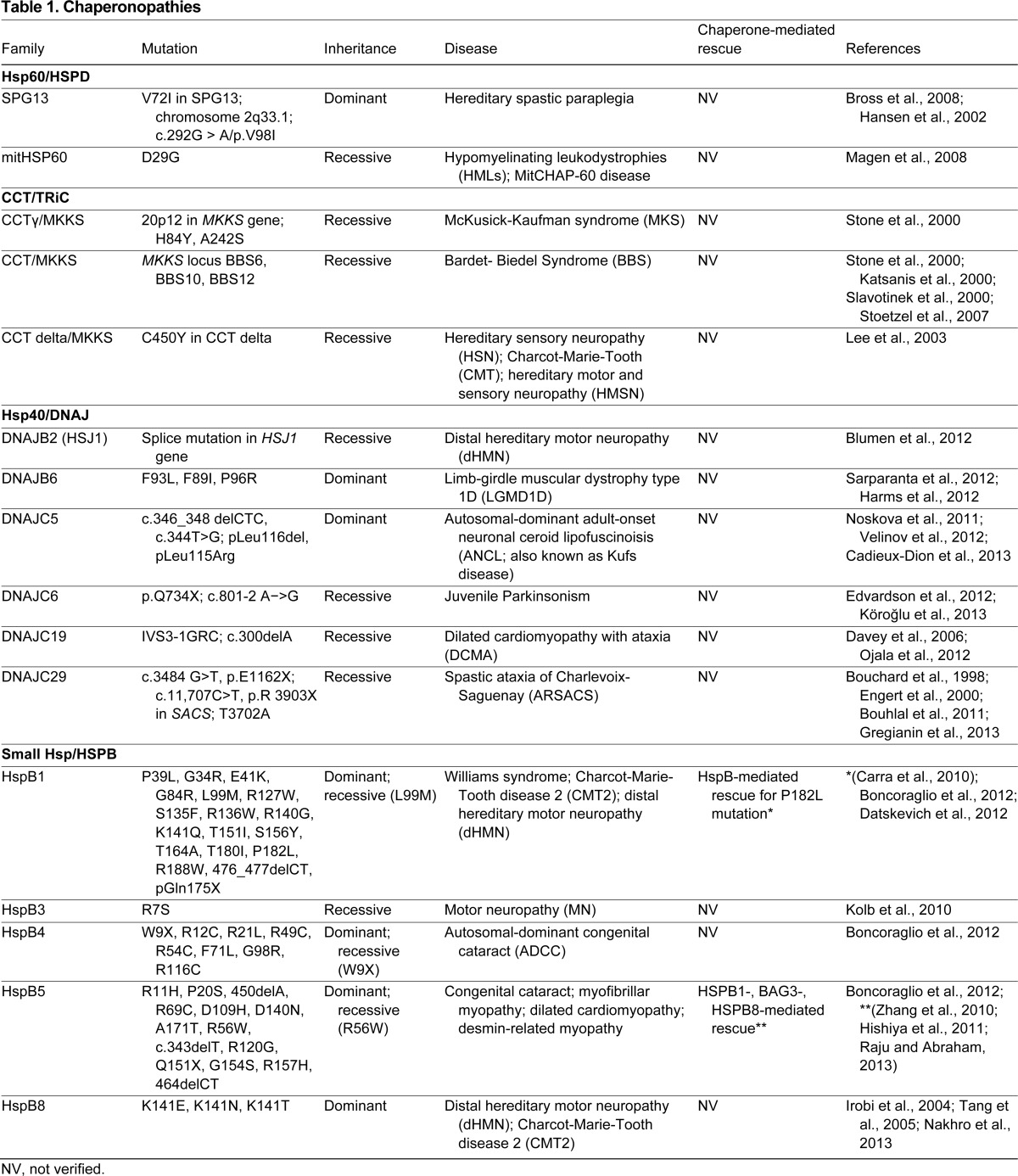
Chaperonopathies

Members of the HSPB, DNAJ and chaperonin families as well as some chaperone cofactors have been implicated in the genetic chaperonopathies described so far (Table 1). No genetic chaperonopathies are associated with the Hsp70/HSPA or Hsp90/HSPC family members, either because of functional redundancy within these families or because they are crucial to the central chaperone machinery such that mutations leading to functional defects are incompatible with life.

**Fig. 3.**
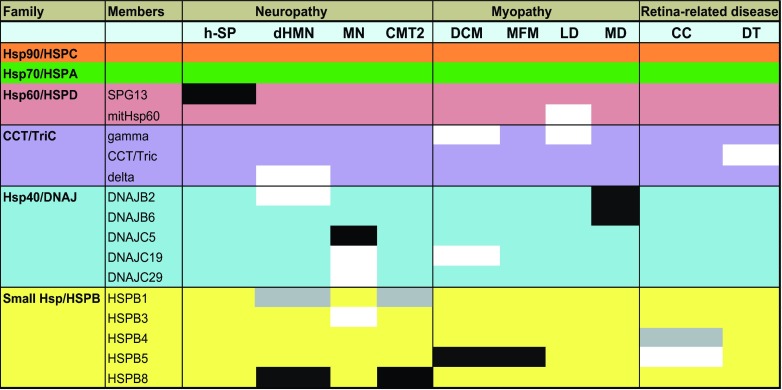
**Overview of chaperonopathies caused by mutations in HSPs.** Mutations that lead to either recessive (white boxes) or dominant (black boxes) chaperonopathies have been described for six ‘families’ of HSP. Each chaperonopathy is categorized as a neuropathy, myopathy or retina-related disease (cataracts). The mutations in HSPs involved in both recessive and dominant diseases have been shaded gray. h-SP, hereditary-spastic paraplegia; dHMN, distal hereditary motor neuropathy; MN, motor neuropathy; CMT2, Charcot-Marie-Tooth disease 2; DCM, dilated cardiomyopathy; MFM, myofibrillar myopathy; LD, leukodystrophy; MD, muscular dystrophy; CC, congenital cataract; DT, dystrophy.

Clinically, genetic chaperonopathies can be categorized into neuropathies [hereditary spastic paraplegia, motor neuropathy, distal hereditary motor neuropathy (dHMN)], myopathies (dilated cardiomyopathy, leukodystrophy, desmin-related myopathy, mitochondrial myopathy, muscular dystrophy) or retina- and eye-lens-related diseases (congenital cataracts) (Macario et al., 2005). Although some chaperonopathies are recessive (and thus probably related to loss of function of the chaperone), most were found to be dominant, as is especially the case for the HSPBs (Table 1). We have labeled or ‘barcoded’ these HSP-associated chaperonopathies depending on the type of disease and mode of inheritance (Fig. 3).

### Hsp60/HSPD- and TRiC/CCT-related chaperonopathies

A mutation in the Hsp60/HSPD chaperone system has been linked to an autosomal-dominant disease known as hereditary spastic paraplegia 13 (SPG13). The disease is characterized by spasticity of lower limbs due to massive degeneration of distal ends of long axons in the spinal cord. The mutation leads to reduced chaperonin activity, which has been attributed to haploid insufficiency due to incorporation of functionally deficient Hsp60/HSPD subunits (Bross et al., 2008; Hansen et al., 2002). Another chaperonopathy involving Hsp60/HSPD is the recessive mitCHAP-60 disease, associated with psychomotor developmental delay, in which mutations lead to entropic destabilization of the Hsp60/HSPD oligomer and cause its premature disassembly. This renders Hsp60/HSPD incapable of fulfilling its normal function, resulting in disease (Parnas et al., 2009).

The Hsp60/HSPD complex resides in mitochondria; however, a comparable eukaryotic chaperonin system known as TRiC (also known as CCT) is present in the cytosol and is mainly involved in tubulin and actin folding. Mutations in TRiC subunits might affect its complex formation and thereby its ability to bind and fold tubulin and actin. Because cytoskeletal integrity is crucial in axonal transport, this might explain why such mutants primarily affect functionality of long axons, thus leading to sensory neuropathies (Lee et al., 2003).

### DNAJ-related chaperonopathies

There are four recessive chaperonopathies associated with members of the DNAJ family (Table 1). The first one involves a DNAJB2 splice mutation that causes dHMN, characterized by muscle weakness of the extremities as a consequence of progressive degeneration of motor neurons in the spinal cord (Blumen et al., 2012). DNAJB2 has several clients and possesses degradation-related functions (Chapple et al., 2004; Westhoff et al., 2005). The DNAJB2 mutant is unable to handle its natural clients, which therefore eventually aggregate and form intracellular inclusions (Blumen et al., 2012).

The second recessive disease caused by mutations in a member of the DNAJ family involves DNAJC29. Mutations in DNAJC29 lead to cerebellar ataxia with peripheral neuropathy, which is referred to as ARSACS. The disease is characterized by dysarthria, distal muscle wasting, foot deformities and truncal ataxia, including the absence of sensory evoked potentials in the lower limbs (Bouchard et al., 1998). Although the normal function of DNAJC29 is not well understood, roles in mitochondrial dynamics and in recruitment of HSPA for the mediation of ataxin-1 degradation have been suggested (Parfitt et al., 2009; Girard et al., 2012). In line with this, a recently identified ARSACS-causing mutation (T3702A) resides in the ubiquitin-binding domain of this protein (Gregianin et al., 2013). However, mutations outside this domain can also lead to disease, so the loss-of-function mechanism remains unclear.

Mutations in DNAJC19 have been identified to cause an autosomal-recessive cardiomyopathy (Davey et al., 2006; Ojala et al., 2012). DNAJC19 normally plays a crucial role in mitochondrial import (Mokranjac et al., 2003), implying that mitochondrial defects underlie the disease.

More recently, mutations in DNAJC6 were found to be associated with juvenile-onset Parkinsonism (Edvardson et al., 2012; Köroğlu et al., 2013). DNAJC6 (also known as auxilin) is a neuron-specific protein that assists Hsc70/HSPA8 in mediating clathrin-coated-vesicle disassembly and thus plays a role in synaptic-vesicle recycling (Ungewickell et al., 1995; Xing et al., 2010). Mutations in DNAJC6 are predicted to lead to a truncated version of the protein, which fails to support Hsc70/HSPA8 in its normal function.

In addition to these recessive diseases, two DNAJ-related chaperonopathies are dominantly inherited (Table 1) and could cause disease either through haploinsufficiency, by dominant-negative effects or via a toxic gain of function. Mutations in DNAJB6, all of which map to a glycine-phenylalanine-rich region, are associated with limb-girdle muscular dystrophy type 1D (LGMD1D). The molecular mechanism underlying the disease has been suggested to involve loss of function resulting in protein accumulations and autophagic pathology in muscle fibers (Harms et al., 2012; Sarparanta et al., 2012). This reduced chaperone function might be due to haploinsufficiency but, because DNAJB6 is present in cells as polydispersed complexes, mutants might also exert dominant-negative effects on the wild-type protein.

Mutations in DNAJC5 cause an autosomal-dominant neurodegenerative disease, named Kufs disease or adult-onset neuronal ceroid lipofuscinosis. Clinical symptoms include dementia, ataxia and speech impairments that worsen over time. Normally, DNAJC5 is found in synaptic vesicles, where it is involved in polymerization of dynamin (Zhang et al., 2012). Dysfunction of DNAJC5 owing to mutations at a crucial lysine position leads to its reduced palmitoylation and hence abnormal sorting and localization of DNAJC5, which colocalized ER and Golgi markers (Nosková et al., 2011). This leads to decreased levels of DNAJC5 in the brain of diseased individuals, meaning that the disease is most likely caused by haploinsufficiency.

### HSPB-related chaperonopathies

Mutations in several members of the HSPB family, irrespective of the member involved, are found in highly conserved amino acid residues or in the α-crystallin domain, which is a characteristic feature of this family of HSPs (Boncoraglio et al., 2012). The α-crystallin domain is required for intra/intermolecular interactions and the stabilization of homo- and hetero-oligomer formations of the HSPB members. Because HSPBs are highly expressed in muscles and have a role in cytoskeleton stability (Tessier et al., 2003; Kampinga and Garrido, 2012), mutations usually affect cellular axonal transport (neurological and sensory disorders) and contractile functions (muscular disorders).

The presence of many of the dominant HSPB mutants in protein aggregates implies that they might have acquired a toxic gain of function similar to the proteins in proteinopathies (Fig. 2). There is indeed biochemical evidence that some mutants, such as the P182L mutant of HSPB1 (Ackerley et al., 2006), R49C and R116C of HSPB4 (Andley et al., 2002; Mackay et al., 2003), and R120G, Q151X and 464delCt of HSPB5 (Bova et al., 1999; Perng et al., 2004; Hayes et al., 2008) are intrinsically unstable and might thus cause disease by forming aggregates (toxic gain of function). However, it must be noted that the presence of HSPBs in aggregates could also be due to a loss of function, reflecting their failed attempt to handle a client with which they subsequently co-aggregate.

Evidence for haploinsufficiency, at least for HSPB1 mutations, is suggested by findings implying that reduced levels of HSPB1 lead to damage in sensory and motor neurons that can be rescued by ectopic expression of HSPB1 (Lewis et al., 1999; Boncoraglio et al., 2012). Partial evidence for haploinsufficiency has also been provided for HSPB8 mutants, which have lost the HSPB8 chaperone-like activity to deal with aggregation-prone polyQ proteins, resulting in Charcot-Marie-Tooth disease (Carra et al., 2008).

Moreover, considering that HSPBs are known to form oligomers with other members of the same family, it is possible that HSPB mutants could affect the function of other HSPBs via dominant-negative effects. For example, HSPB8 mutants have an abnormally high affinity for endogenous HSPB1, thus potentially impairing HSPB1 or HSPB1-HSPB8 complex function (Irobi et al., 2004). Similarly, certain HSPB1 mutants affect endogenous HSPB8, leading to loss of HSPB8-HSPB1 complex formation (Fontaine et al., 2006). Furthermore, abnormal interaction of the R116C-HSPB4 mutant with HSPB5 and HSPB1 has been reported (Fu and Liang, 2002; Fu and Liang, 2003).

Therefore, not only a toxic gain of function (aggregation) might be responsible for HSPB-related chaperonopathies, but also a loss of function, which could either be direct, due to the mutation, or indirect, due to sequestration of wild-type HSPBs by the mutated HSPB forms. In addition, alteration of HSPB oligomerization properties and interactions with other HSPB members and/or haploinsufficiency might play a role in HSPB-related chaperonopathies.

### Chaperone intervention to rescue chaperonopathies

Different HSPs have a role in anti-aggregation of various proteinopathies (Fig. 2). However, whether other HSPs might be able to rescue chaperonopathies has been scarcely studied. A few reports suggest that this could indeed be possible. Firstly, aggregation caused by some HSPB5 mutants is prevented by overexpression of wild-type HSPB1 (Zhang et al., 2010; Raju and Abraham, 2013), BAG3 (Hishiya et al., 2011) and wild-type HSPB8 (Chávez Zobel et al., 2003). Secondly, aggregation associated with the expression of the P182L-HSPB1 mutant in cell models was significantly reduced by the overexpression of wild-type HSPB8 (Carra et al., 2010). Whether such rescues are due to prevention of the formation of toxic aggregates containing mutant HSPB or whether they reflect compensation of loss of HSPB functions remains to be elucidated.

### Conclusions and future perspectives

The existence of different ‘barcodes’ for the rescue of specific aggregation diseases suggests that, although loss of protein homeostasis with aging might contribute to disease initiation (e.g. by HSF-1 abrogation, restoring general protein homeostasis or components thereof), boosting HSF-1 activity is usually insufficient for long-term protection in most dominantly inherited proteinopathies. Chronic expression of these aggregation-prone proteins in fact often does not trigger activation of the HSR until late in disease. By then, aggregates might have already sequestered chaperones and thereby disturbed normal protein homeostasis, resulting in cell death. In earlier stages of disease, protein aggregates could already affect neuronal and muscular cell function (even without causing cell death) by altering functions such as axonal transport, organelle dynamics and plasma-membrane-receptor function, without directly impairing protein homeostasis. It has been shown in several mouse models for HD that reversible functional impairments precede neuronal cell loss (Yamamoto et al., 2000). However, in cellular and simple animal models, such functional defects could be missed, and cell-death-related effects (including disturbances in protein homeostasis) might prevail, which would explain the observed rescue by the activation of the HSR or by the overexpression of its individual components. However, these HSR-related effects usually do not coincide with aggregate prevention and therefore do not lead to significant long-term effects in mammalian animal models.

The human genome encodes many HSP members that are not regulated by the acute HSR. Although not yet studied intensively, our review clearly shows that some of these ‘non-canonical’ members can specifically rescue aggregation caused by the distinct proteinopathies, some of which have now also been demonstrated to be effective in mouse models. Interestingly, several of these non-canonical HSPs also cause chaperonopathies if mutated (DNAJB2, DNAJB6, HSPB8). This not only indicates that these HSPs have essential PQC functions, but also suggests that their effects on proteinopathies might not be an artifact of their overexpression, but rather reflect an augmentation of their natural function.

A potential worry in all HSP-overexpression or -boosting studies is that it leads to network adaptations (which would annihilate long-term effectiveness) or to multiple side effects, including enhancement of carcinogenesis, as was demonstrated for the manipulation of HSF-1 activity (Mendillo et al., 2012). Although network adaptations are to be expected upon manipulation of the driving forces of chaperone machinery (e.g. Hsp90/HSPC or Hsp70/HSPA), such effects might be less likely for those components that only steer the specificity of these machines (e.g. HSPBs or DNAJs). Although we found no evidence for effects of DNAJB6 on the chaperone network (our unpublished results), it remains important to further investigate whether long-term overexpression of DNAJB6 or other proteinopathy-rescuing HSPs might have side effects.

Finally, only limited comparative data on the potential rescue of the non-HSR-regulated HSPs and the various proteinopathies or chaperonopathies are available. There still might be many currently unknown suppressors of specific diseases to be uncovered, which would further barcode these diseases. This would not only help to pinpoint therapeutic targets for intervention, but would also help with understanding differences and similarities between the toxic mechanisms underlying the various proteinopathies.
